# Influence of *IL17A* polymorphisms on the aberrant methylation of *DAPK* and *CDH1* in non-cancerous gastric mucosa

**DOI:** 10.1186/1471-2350-13-59

**Published:** 2012-07-24

**Authors:** Tomiyasu Arisawa, Tomomitsu Tahara, Mikihiro Tsutsumi, Tomoyuki Shibata

**Affiliations:** 1 Department of Gastroenterology, Kanazawa Medical University, 1-1, Daigaku, Uchinada-machi, Ishikawa, 920-0293, Japan; 2 Department of Gatroenterology, Fujita Health University, 1-98, Dengakugakubo, Kutsukake-cho, Toyoake, 470-1192, Japan

**Keywords:** *IL17A*, Aberrant DNA methylation, *DAPK*, *CDH1*

## Abstract

**Background:**

CpG island aberrant methylation is shown to be an important mechanism in gene silencing. The important role of IL-17 in inflammatory response to *H. pylori* colonization has been indicated. We investigated the influence of *IL17A* polymorphisms, -197 G > A (rs2275913) and *1249 C > T (rs3748067), on the methylation of *DAPK* and *CDH1*.

**Methods:**

Gastric mucosal samples were obtained from 401 subjects without malignancies. Methylation status of gene was determined by MSP. The genotyping of *IL17A* was performed by PCR-SSCP.

**Results:**

Methylations of *DAPK* and *CDH1* were seen in 196 and 149 of all 401 subjects, respectively. Overall, *1249 T carrier was associated with a decreased risk for *DAPK* methylation, whereas -197 G > A was not. In the subjects older than 60 years old, *1249 T carrier was more strongly associated with gene methylation and -197 A carrier tended to be associated with an increased risk for *CDH1* methylation. When evaluating by inflammation promoting haplotype (-197 mutant carrier with *1249 homozygote), this haplotype had a more strongly increased risk for both *DAPK* and *CDH1* methylations in comparatively older subjects. Both atrophy and metaplasia scores were significantly increased with age in -197 A carrier or *1249 CC homozygote, whereas were not in -197 GG homozygote or *1249 T carrier. PG I/II ratio was more significantly decreased in -197 A carrier than in GG homozygote under influence of *H. pylori* infection.

**Conclusions:**

In -197 A allele carrier with *1249 CC homozygote, the methylations of both *DAPK* and *CDH1* may be increased gradually, but more rapidly than the other genotypes, with age and altered gastric mucosal structure induced by *H. pylori* infection.

## Background

Interleukin-17 (IL-17) is a relatively newly described cytokine that bridges the adaptive and innate immune systems. IL-17A is a cytokine responsible for the pathogenic activity of the Th17 cells [1], a distinct lineage of CD4+ effector cells [2]. IL-17A induces multiple proinflammatory mediators, including chemokines, cytokines, and metalloproteinases, from epithelial and fibroblast cells [3]. An enhanced expression of IL-17 has been also documented and implicated in the pathogenesis of immune-mediated diseases, such as rheumatoid arthritis, multiple sclerosis, and psoriasis [4]. In addition, IL-17 has the ability to stimulate IL-8 production in both epithelial cells and macrophages [5,6], raising the possibility that this cytokine may play an important role in the recruitment of inflammatory cells during bacterial infections. At first, Lussa et al. reported that bio-active Il-17A production was increased during *Helicobacter pylori* (*H. pylori*) infection, suggesting the possibility that this cytokine may play an important role in the *H.pylori*-induced inflammatory response [7]. Thereafter, several studies reported that IL-17 stimulates IL-8 release by gastric epithelial cells and facilitates the chemotaxis of neutrophils through an IL-8-dependent mechanism, and contributes to the enhancement of IL-8 levels in *H. pylori*-colonized gastric mucosa [8-10].

On the other hand, gastric cancer is one of the most common cancers worldwide [11,12], but the etiology of this tumor remains unclear. *H. pylori* infection is now accepted as a crucial event in the development of peptic ulcer disease and atrophic gastritis, and it is implicated in the development of gastric carcinoma, especially not located in cardia [13-15]. Several cancers, including gastric tumors, show methylations of multiple genes including E-cadherin gene (*CDH1*)*,* death-associated protein kinase gene (*DAPK*) and *CDKN2A*[16,17]. Some genes are also methylated in non-neoplastic tissues with aging, and this alteration is known as age-related methylation [18,19]. In addition, it has also shown that gene methylation may be present in chronic inflammation of various tissues [20,21]. In gastric mucosa, it was indicated that methylation of CpG island was induced by *H. pylori* infection in non-cancerous mucosa [22,23] and considered as the precancerous conditions in gastric carcinogenesis [24]. Among several genes, *DAPK* and *CDH1*, as well as *CDKN2A*, are frequently methylated in non-neoplastic gastric mucosa in relation to age, *H. pylori* infection, histological degree of gastritis, and gastric carcinogenesis [22,25,26].

Recently, we have reported that the IL-17A gene (*IL17A*) polymorphism (rs2275913 G > A) and IL-17 F gene (*IL17F*) polymorphism (rs763780 T > C) are closely associated with the susceptibility to gastric carcinogenesis [27], as well as ulcerative colitis [28]. Thereafter, several studies revealed the association between *IL17A* rs2275913 G > A and rheumatoid arthritis, gastric carcinogenesis and asthma [29-31]. The rs2275913 (G/A), located at a position -197 from the starting codon of *IL17A*, may regulate the expression of mRNA. In addition, there is a polymorphism rs3748067 (*1249 C > T) in *IL17A* 3’-UTR, targeted by some microRNAs. We hypothesize that these *IL17A* gene polymorphisms may influence the development of aberrant DNA mathylation of gastric mucosa.

In the present study, we investigated the association between polymorphisms of *IL17A*, rs2275913 (-197 G > A) and rs3748067 (*1249 C > T), and the aberrant DNA methylation of *DAPK* and *CDH1* in non-cancerous gastric epithelium.

## Methods

### Clinical samples

The studied population comprised 401 subjects without gastric malignancies recruiting the Endoscopy Center of Fujita Health University Hospital or Kanazawa Medical University Hospital. In HapMap-JPT, the frequency of *IL17A -*197 A allele frequency was 45.3%. We assume that a 20% decrease in the prevalence of an allelic frequency would be clinical relevance (unmethylated group: 40% vs. methylated group: 50%). Assuming an alpha value = 0.05 and a power = 0.80, at least 200 unmethylated subjects and 200 methylated subjects would be sufficient to identify a clinical relevant difference. Accordingly, 400 subjects would be clinical relevance for the study. All subjects underwent upper endoscopy with one or two biopsy specimens from non-cancerous mucosa in antrum. Parts of each specimen was fixed in 10% buffered formalin and embedded in paraffin, while the other part was immediately frozen and stored at -80°C. Later, Genomic DNA was isolated from frozen specimens using proteinase K. A specimen from the subjects who consented to only one biopsy was not used for the histological evaluation.

The subjects without methylation of both *DAPK* and *CDH1* were classified into non CIHM (CpG island high methylated) group, whereas the others except non CIHM were classified into CIHM group. Furthermore, the subjects were divided into 2 groups by genotype as follows:

HR (high risk) group: *IL17A -*197 A carrier with *1249 CC genotype

LR (low risk) group: the subjects except HR group

The Ethics Committees of the Fujita Health University and Kanazawa Medical University approved the protocol, and prior, written informed consent was obtained from all participating subjects.

### Bisulfate modification and Methylation-Specific PCR (MSP)

To examine DNA methylation, genomic DNA was treated with sodium bisulfite using BislFast DNA Modification Kit for Methylated DNA Detection (TOYOBO, Co., Ltd., Osaka, Japan). MSP for *DAPK* and *CDH1* were carried out using the methods reported by Katzenellenbogen et al. [32] and Herman et al. [33], respectively. In brief, MSP reactions were carried out with primer pairs described below, using EX Taq HS (Takara Bio, Inc., Shiga, Japan).

Primer pairs:

DAPK: methylated forward; 5’-ggatagtcggatcgagttaacgtc-3’,reverse; 5’-ccctcccaaacgccga-3’,

DAPK: unmethylated forward; 5’-ggaggatagttggattgagttaatgtt-3’,reverse; 5’-caaatccctcccaaacaccaa-3’

CDH1: methylated forward; 5’-ttaggttagagggttatcgcgt-3’,reverse; 5’-taactaaaaattcacctaccgac-3’,

CDH1: unmethylated forward; 5’-taattttaggttagagggttattgt-3’,reverse; 5'-cacaaccaatcaacaacaca-3’,

An annealing temperature and times were determined using DNA from peripheral blood of a young individual without *H. pylori* infection and DNA methylated with SssI methylase (NEW ENGLAND BioLabs Inc., Beverly, MA). The MSP was carried out in a volume of 20μL containing 0.1 μg of bislufite-modificated DNA. The bands of MSP were detected by electrophoresis in 3.0% agarose gels stained with ethdium bromide. Hypermethylation was defined as the presence of a positive methylation band showing signals approximately equal to or greater than that of the size marker (10 ng/ μL: 100 bp DNA Ladder, Takara Bio, Inc., Shiga, Japan) regardless of the presence of unmethylated bands.

### Genotyping of polymorphisms

The DNA isolated from biopsy specimens or peripheral blood was used. Polymorphism was genotyped by PCR-SSCP method as reported previously [27,28]. To detect *IL-17A* *1249 C > T using the primer pairs (1249F: 5’-cccctcagagatcaacagaccaaca-3’and 1249R: 5’-gcgaaaatggttacgatgtgaaacttg-3’), PCR was carried out in a volume of 20 μL containing 0.1 μg of genomic DNA. The DNA was denatured at 95°C for 3 minutes, followed by 35 cycles at 95°C for 30 seconds, 52°C for 40 seconds, and 72°C for 45 seconds, with final extension at 72°C for 5 minutes. Thereafter, 2 μL of the PCR product was denatured with 10 μL of formamide (Sigma-Aldrich Co., St. Louis, USA) at 90°C for 5 minutes. SSCP was carried out at 18°C using a GenePhor DNA separation system with GeneGel Excel 12.5/24 (Amersham Biosciences Corp., USA), after which the denatured single strand DNA bands were detected using a DNA Silver Staining Kit (Amersham Biosciences Corp.).

To detect IL-17A -197 G > A, using the primer pairs (IL17AF: 5’-aacaagtaagaatgaaaagaggacatggt-3’ and IL17AR: 5’-cccccaatgaggtcatagaagaatc-3’), PCR was carried out in a volume of 20 μL containing 0.1 μg of genomic DNA. The DNA was denatured at 96°C for 90 s, followed by 35 cycles at 96°C for 15 s, 58°C for 30 s, and 72°C for 45 s, with final extension at 72°C for 3 min. Thereafter, SSCP was carried out at 6°C as a same manner described above.

### Histological evaluation

In 286 of 401 subjects, the severity of chronic gastritis was classified according to the updated Sydney system [34] by a pathologist who had no access to any clinical information.

### Serological evaluation

The pepsinogen (PG) I/II ratio was calculated based on the data of the serum PG I and PG II levels measured by radioimmunoassay in 74 of 401 subjects. A PG I/II ratio that showed a decrease in proportion to the severity of gastric mucosal atrophy was used as a marker of atrophic gastritis [35,36].

### Statistical analysis

The data were expressed as mean ± SD. Mean ages between 2 groups was compared by Student’s *t-*test. The ratio of *H. pylori* infection status and male/female was compared by Fisher’s exact test. Allele and genotype frequencies were calculated by direct counting. The allele counts were also compared by a Fisher’s exact test. The strength of association between allele frequencies and the disease was assessed by calculating the odds ratio (OR) and 95% confidence intervals (CI) by logistic regression analysis. Adjusted ORs were calculated after adjustment for age, gender and *H. pylori* infection status. Each updated Sydney system score and PG I/II ratio between 2 groups were compared by Mann Whitney U-test. The relationship between age and updated Sydney system score was assessed by ANCOVA. When setting α = 0.05, the β value was calculated by Post-hoc analysis. For all analyses, the level of significance was set at *p* < 0.05.

## Results

### Subjects and genotype

The characteristics of subjects were summarized in Table 1. *H. pylori* positive ratio was significantly higher in CIHM group than in non CIHM group. The distribution of -197 G > A genotype in non CIHM group was 65GG, 74GA and 14AA. It was in the Hardy-Weinberg equilibrium (p = 0.36). That of *1249 C > T was 124cm^3^, 20CT and 9TT, which was not in the Hardy-Weinberg equilibrium. The distribution of -197 G > A genotype was not different among two groups. However, the frequencies of *1249 minor allele was significantly lower in CIHM group than non CIHM group (p = 0.023, 1-βpower = 0.636).

**Table 1:** Characteristics of the subjects and frequency of genotypes

	**Total**	**non CIHM**	**CIHM**	p value*
number of subjects	401	153	248	
mean age ± SD	60.0 ± 13.8	60.2 ± 13.7	59.9 ± 13.9	NS
male : female	230 : 171	87 : 66	143 : 105	NS
*H. pylori* positive rate	241/401	71/153	170/248	<0.0001
-197 G > A				
GG	155	65	90	
GA	204	74	130	
AA	41	14	27	
(unknown)	1	0	1	
A allele frequency	35.8%	33.3%	37.2%	NS
*1249 C > T				
CC	340	124	216	0.058^a^
CT	42	20	22	
TT	16	9	7	
(unknown)	3	0	3	
T allele frequency	9.3%	12.4%	7.3%	0.023

non CIHM: neither *DAPK* nor *CDH1* were methylated.

CIHM: both or either *DAPK* or *CDH1*was methyaled.

*: unmethylated vs. methylated.

a: frequency of *1249 CC genotype.

### Distributions of IL17A genotypes and gene mathylations

The *H. pylori* positive ratio was significantly higher in methylated group than in unmethylated group of *DAPK* or *CDH1* (Table 2). The distribution of -197 G > A genotype was not different among two groups of both genes, whereas the frequency of *1249 CC homozygote was significantly higher in methylated group than in unmethylated group of *DAPK* (p = 0.023). In addition, the minor allele frequency was significantly lower in methylated group than in unmethylated group of *DAPK* (*p* = 0.020, 1-βpower = 0.641). However, the distribution of *1249 C > T genotype was not different among two groups of *CDH1*.

**Table 2:** Distributions of IL17A genotypes and gene mathylations

	**DAPK**			**CDH1**		
	**Unmethylated**	**methylated**	p value*	**unmethylated**	**methylated**	p value*
number of subjects	205	196		252	149	
mean age ± SD	59.3 ± 13.6	60.8 ± 13.9	NS	60.1 ± 14.1	59.9 ± 13.2	NS
male : female	118 : 87	112 : 84	NS	145 : 107	85 : 64	NS
*H. pylori* positive rate	104/205	137/196	<0.0001	131/252	110/149	<0.0001
-197 G > A						
GG	86	69	98	57		
GA	99	105	128	76		
AA	19	22	26	15		
(unknown)	1	0	0	1		
A allele frequency	33.6%	38.0%	NS	35.7%	35.8%	NS
*1249 C > T						
CC	167	173	0.023^a^	210	130	
CT	28	14		28	14	
TT	10	6	11	5		
(unknown)	0	3		3	0	
T allele frequency11.	7%	6.7%	0.020	10.0%	8.2% NS	

*: unmethylated vs. methylated.

a: frequency of *1249 CC genotype.

### Risk of IL17A polymorphisms for the gene methylation

The -197 G > A was not associated with CIHM and was not associated with each *DAPK* and *CDH1* methylations (Table 3). On the other hand, *1249 C > T tended to be associated with CIHM (OR, 0.611; 95%CI, 0.343-1.09; p = 0.096, Table 4). Meanwhile, *1249 mutant carrier had a decreased risk for the development of *DAPK* methylation (OR, 0.513; 95%CI, 0.282-0.933; p = 0.028), and had no significant risk for the development of *CDH1* methylation (p = 0.62, Table 4).

**Table 3:** Association between IL17A -197 G > A polymorphism and gene methylation

**CIHM (*DAPK*or*CDH1*)**	**GG**	**GA**	**AA**	**unknown**	**A carrier vs. GG; OR (95%CI)**	**p value**
unmethylated (153)	65	74	14	0	reference	-
methylated (248)	90	130	27	1	1.33 (0.870-2.04)	0.19
*DAPK*						
unmethylated (205)	86	99	19	1	reference	-
methylated (196)	69	105	22	0	1.37 (0.907-2.08)	0.13
*CDH1*						
unmethylated (252)	98	128	26	0	reference	-
methylated (149)	57	76	15	1	1.04 (0.676-1.60)	0.86

by logistic regression analysis after adjustment for age, gender and *H. pylori* infection status.

( ): number of subjects.

**Table 4:** Association between IL17A *1249 C > T polymorphism and gene methylation

**CIHM (*DAPK*or*CDH1)***	**CC**	**CT**	**TT**	**unknown**	**T carrier vs. CC; OR (95%CI)**	**p value**
unmethylated (153)	124	20	9	0	reference	-
methylated (248)	216	22	7	3	0.611 (0.343-1.09)	0.096
*DAPK*						
unmethylated (205)	167	28	10	0	reference	-
methylated (196)	173	14	6	3	0.513 (0.282-0.933)	0.028
*CDH1*						
unmethylated (252)	210	28	11	3	reference	-
methylated (149)	130	14	5	0	0.856 (0.466-1.57)	0.62

by logistic regression analysis after adjustment for age, gender and *H. pylori* infection status.

( ): number of subjects.

Because mean age of our subjects was approximately 60 years old and gene methylation is increased with aging, we made further assessment in the subjects older than 60 years old. Then, -197 A carrier had an increased risk for the development of CIHM (OR, 1.80; 95%CI, 1.01-3.19; p = 0.046), conversely *1249 T carrier had a decreased risk (OR, 0.463; 95%CI, 0.224-0.959; p = 0.038, Table 5). When assessing the risk for each *DAPK* and *CDH1* methylation, -197 A carrier tended to have an increased risk for the development of *CDH1* methylation (p = 0.068), whereas *1249 T carrier had a decreased risk for the development of *DAPK* methylation (OR, 0.427; 95%CI, 0.204-0.893; p = 0.024).

**Table 5:** Risk of IL17A plymorphisms for gene methylation in the subjects older than 60 years old

**CIHM (*DAPK*or*CDH1)***						
* IL17A* -197 G > A	GG	GA	AA	unknown	A carrier vs. GG; OR (95%CI)	p value
unmethylated (83)	38	39	6	0	reference	-
methylated (143)	46	79	18	0	1.80 (1.01-3.19)	0.046
* IL17A* *1249 C > T	CC	CT	TT	unknown	T carrier vs. CC; OR (95%CI)	p value
unmethylated (83)	63	15	5	0	reference	-
methylated (143)	124	13	5	1	0.463 (0.224-0.959)	0.038
*DAPK*						
* IL17A* -197 G > A	GG	GA	AA	unknown	A carrier vs. GG; OR (95%CI)	p value
unmethylated (103)	44	50	9	0	reference	-
methylated (123)	40	68	15	0	1.57 (0.897-2.75)	0.11
* IL17A* *1249 C > T	CC	CT	TT	unknown	T carrier vs. CC; OR (95%CI)	p value
unmethylated (103)	79	19	5	0	reference	-
methylated (123)	108	9	5	1	0.427 (0.204-0.893)	0.024
*CDH1*						
* IL17A* -197 G > A	GG	GA	AA	unknown	A carrier vs. GG; OR (95%CI)	p value
unmethylated (145)	60	71	14	0	reference	-
methylated (81)	24	47	10	0	1.76 (0.958-3.22)	0.068
* IL17A* *1249 C > T	CC	CT	TT	unknown	T carrier vs. CC; OR (95%CI)	p value
unmethylated (145)	116	22	6	1	reference	-
methylated (81)	71	6	4	0	0.590 (0.263-1.32)	0.20

by logistic regression analysis after adjustment for age, gender and *H. pylori* infection status.

( ): number of subjects.

When assessing the risk of HR (-197 A carrier with *1249 CC genotype), HR tended to have an increased risk for CIHM (p = 0.081), and had a significantly increased risk for the development of *DAPK* methytation (OR, 1.57; 95%CI, 1.04-2.35; p = 0.031, Table 6). In the subjects older than 60 years old, HR had an increased risk for CIHM (OR, 1.93; 95%CI, 1.10-3.39; p = 0.023), and had an increased risk for the development of each *DAPK* methylation and *CDH1* methylation (OR, 1.91; 95%CI, 1.10-3.30; p = 0.021 and OR, 2.01; 95%CI, 1.12-3.62; p = 0.020, respectively). In addition, HR had a more increased risk for the development of the methylation of both two genes (OR, 2.42; 95%CI, 1.25-4.68; p = 0.0087, 1-βpower = 0.709).

**Table 6:** Risk of rs2275913 and rs3748067 for gene methylations

**CIHM (*DAPK*or*CDH1)***					
over all	HR	LR	unknown	HR vs. LR; OR (95%CI)	p value
unmethylated (153)	76	77	0	reference	-
methylated (248)	144	101	3	1.45 (0.955-2.20)	0.081
60 = <					
unmethylated (83)	38	45	0	reference	-
methylated (143)	89	54	0	1.93 (1.10-3.39)	0.023
*DAPK*					
over all	HR	LR	unknown	HR vs. LR; OR (95%CI)	p value
unmethylated (205)	102	102	1	reference	-
methylated (196)	118	76	2	1.57 (1.04-2.35)	0.031
60 = <					
unmethylated (103)	49	54	0	reference	-
methylated (123)	78	45	0	1.91 (1.10-3.30)	0.021
*CDH1*					
over all	HR	LR	unknown	HR vs. LR; OR (95%CI)	p value
unmethylated (252)	134	116	2	reference	-
methylated (149)	86	62	1	1.20 (0.787-1.83)	0.40
60 = <					
unmethylated (145)	73	72	0	reference	-
methylated (81)	54	27	0	2.01 (1.12-3.62)	0.020
*DAPK* and *CDH1*					
over all	HR	LR	unknown	HR vs. LR; OR (95%CI)	p value
unmethylated (304)	160	141	3	reference	-
methylated (97)	60	37	0	1.44 (0.890-2.34)	0.14
60 = <					
unmethylated (165)	84	81	0	reference	-
methylated (61)	43	18	0	2.42 (1.25-4.68)	0.0087

HR: -197 A carrier with*1249 CC genotype, LR: the others by logistic regression analysis after adjustment for age, gender and *H. pylori* infection status.

( ): number of subjects.

### Association between IL17A polymorphisms and histological or serological gastric mucosal atrophy

**Figure 1:**
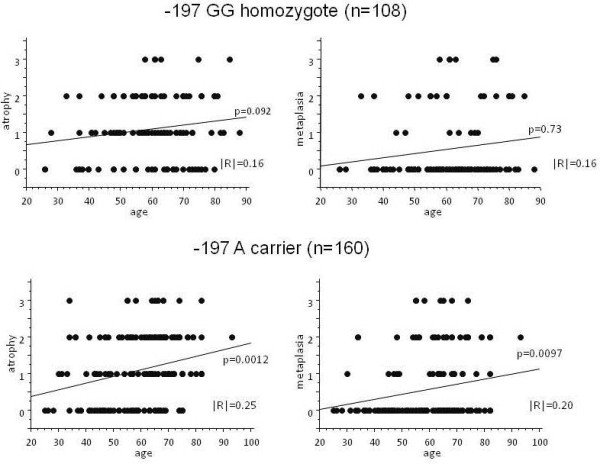
**Association between gastric mucosal atrophy and IL17A -197 G > A polymorphism.** Both atrophy and metaplasia scores were significantly correlated to the age in A carrier (mutant carrier), whereas not in GG homozygote (wild homozygote).

**Figure 2:**
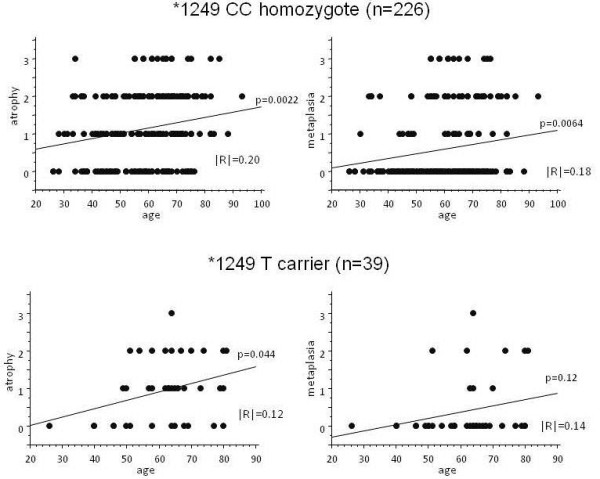
Association between gastric mucosal atrophy and IL17A *1249 C > T polymorphism. Both atrophy and metaplasia scores were significantly correlated to the age in CC homozygote (wild homozygote), whereas only atrophy score was correlated to the age, but weakly, in T carrier (mutant carrier).

Both atrophy and metaplasia scores were strongly correlated to the age in -197 A carrier (p = 0.0012 and 0.0097 by ANOVA, respectively, Figure 1), whereas there was no correlation between age and both scores in GG homozygote (p = 0.092 and p = 0.73, respectively). Both scores were also strongly correlated to the age in *1249 CC homozygote (p = 0.0022 and 0.0064, respectively, Figure 2), whereas only atrophy score was weakly correlated to the age in T carrier (p = 0.044).

**Figure 3:**
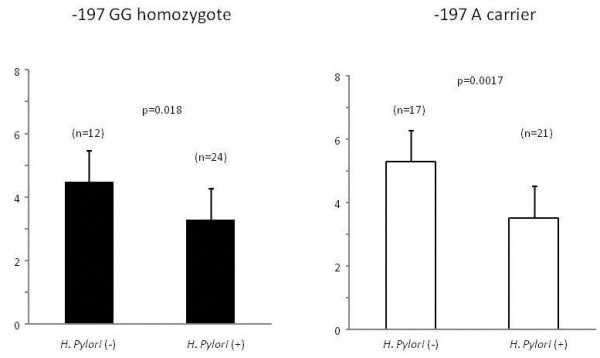
**Change of PG I/II ratio under the influence of H. pylori infection by IL17A -197 G > A genotype.** PG I/II ratio was significantly lower in *H. pylori* positive subjects than negative subjects in both -197 A carrier and GG homozygote, but in the former more strongly significant.

PG I/II ratio was significantly lower in *H. pylori* positive subjects than negative subjects in both -197 A carrier and GG homozygote, but in the former more strongly significant (p = 0.0017 and 0.018, respectively, Figure 3). The association between *1249 C > T and PG I/II ratio could not be evaluated, because the number of T carrier assessed was very small.

## Discussion

Previously, we found no association between *IL17A* -197 G > A and methylation of both *DAPK* and *CDH1*[37]. In the present study with a large number of subjects, we confirm these results. However, our present study showed that *IL17A -*197 A carrier tended to have an increased risk for the development of methylation, especially of *CDH1*, in the subjects older than 60 years old. In addition, the association of *1249 C > T located in 3’-UTR was also investigated in the present study. The *1249 T carrier was an increased risk factor for *DAPK* methylation and more strongly associated with the increased risk for gene methylation in comparatively older subjects (60 years = <). Over all, *1249 C > T seemed to be more closely associated with gene methylation than -197 G > A. When assessing the association of *IL17A* haplotype, -197 mutant (A allele) carrier with *1249 wild homo alleles (CC homozygote) had a more strongly increased risk for the development of gene methylation. Because allele frequency was less than prior assumptions, study population has been rather small. Therefore, the effect of type II error cannot be excluded in the assessment of allelic or genotype association of polymorphisms with gene methylation. This is one of the major limitations in our study.

Although the mechanisms of gene methylation are unknown, several factors may contribute to this methylation, such as exogenous carcinogens, generated reactive oxygen and host genetic differences [38]. One of the most important factors causing gene methylation in stomach is *H. pylori* infection [17], which first induces chronic superficial gastritis, which can progress to chronic atrophic gastritis, intestinal metaplasia, and dysplasia that leads toward gastric carcinoma [39]. In facts, the methylation of certain genes in non-neoplastic gastric mucosa correlate with *H. pylori* related histological or serological severity of gastritis [26] and gastric cancer occurrence [23,40,41]. IL-17A promotes the gastric mucosal inflammation via increased IL-8 production [8-10]. In addition, IL-17A induces increased levels of reactive oxygen species, which is one of the major inflammatory mediators [42]. Methylation of cytosine residues in DNA can be greatly influenced by hydroxyl free radical adducts on adjacent guanine residues [43]. IL-17A may contribute to increased gene methylation in *H. pylori*-induced gastric inflamed mucosa through these mechanisms.

Recently, it has been also reported that *IL17A -*197 AA homozygote is associated with several asthma-related traits and confers genetic susceptibility to childhood asthma in Chinese [31]. Furthermore, in our present study, both atrophy and metaplasia scores increased with age in -197 A carrier, whereas did not in GG homozygote. PG I/II ratio was more significantly decreased by *H. pylori* infection in A carrier than GG homozygote. In general, gastric mucosal atrophy develops as a result of severe inflammation that continued for a long term [39]. These suggest that *IL17A -*197 A allele may promote the inflammation. However, we found no significant difference of inflammation score among genotypes (data not shown). The degree of inflammation seems to fluctuate according to the inflammatory environment and is decreased with the progression of gastric mucosal atrophy. Therefore, it may be difficult to determine the degree of inflammation at one point. On the other hand, there is the possibility that *IL17A* polymorphisms may be associated with gastric mucosal atrophy by the mechanism other than promoting the inflammation. It is interesting to understand the effect of -197 A allele on the expression of IL-17A. Chen et al. demonstrated that *IL17A* -197 G > A was not related to PHA induced IL-17 production in PBMCs [31]. However, many kinds and subtypes of cells existed in PBMCs and the networks of regulating immune response and cytokine production were complicated. In addition, IL-17A seemed to have dual actions, inflammatory or protective, depending of the co-expression of other cytokines [44]. Therefore, the function of *IL17A -*197 G > A in the inflammation is still well unknown.

On the other hand, to our best knowledge, there have been no reports of the association between *IL17A* *1249 C > T, located in 3’-UTR, and human disorders. In general, microRNAs post-transcriptionally regulate the expression of mRNA through the binding to 3’-UTR of target genes. Therefore, altered conformation of 3’-UTR may affect the binding of microRNAs to mRNA. In our present study, *1249 mutant carrier had a decreased risk for gene methylation, especially *DAPK*, in comparative older subjects. Moreover, it seemed that the repressor activity of *1249 T allele took part in the inflammations more strongly than the stimulatory effects of -197 A allele. In addition, both atrophy and metaplasia scores increased with age in *1249 wild homozygote (CC genotype), whereas only atrophy score weakly correlated to the age in mutant carrier. These suggest that *1249 T allele may protect the inflammation. That is, we speculate that *IL17A -*197 mutant allele may promote and *1249 mutant allele may protect the inflammation.

Based on these results, we evaluated the association of HR, as an inflammation promoting haplotype, (-197 mutant carrier with *1249 wild homozygote) with gene methylation. In the subjects older than 60 years old, HR was more strongly associated with the increased risk for both *DAPK* and *CDH1* methylation, although overall with only *DAPK* methylation. This suggests that both -197 G > A and *1249 C > T may affect on the expression or function of IL-17A coordinately. The reason why significant differences were observed in comparatively older subjects may be because gene methylation is age-related change and/or because methylation depends on severity and periods of chronic inflammation. We speculate that this difference becomes clear after gene methylation progressed at the old age.

## Conclusions

In conclusions, the inflammation promoting allele of *IL17A* -197 G > A (rs2275913) and *1249 C > T (rs3748067) was significantly associated with the increased risk for the development of gene mathylations in non-cancerous gastric mucosa. That is, in -197 A allele carrier with *1249 CC homozygote, the methylation of *DAPK* and *CDH1* is increased gradually, but more rapidly, with age and altered gastric mucosal structure induced by *H. pylori* infection than in the others.

## Abbreviations

ANOVA: Analysis of variance; *CDH1*: E-cadherin gene; *CDKN2A*: Cyclin-dependent kinase inhibitor 2A gene; CI: Confidence interval; CIHM: CpG island high methylated; *DAPK*: Death-associated protein kinase gene; *H. pylori*: Helicobacter pylori; IL: Interleukin; *IL17A*: Interleukin 17A gene; MSP: Methylation-specific PCR; OR: Odds ratio; PBMC: Peripheral blood mononuclear cell; PG: Pepsinogen; PHA: Phytohemagglutinin; SSCP: Single Strand Conformation Polymorphism; SD: Standard deviation; UTR: Untranslated region.

## Competing interests

The authors declare that they have no competing interests.

## Authors’ contributions

TA analyzed the data, wrote the paper and was responsible for the conception of the study and designed the study. TT participated in the design of the study. MT and TS obtained the samples and the data. All authors approved of the final manuscript prior to submission.

## Pre-publication history

The pre-publication history for this paper can be accessed here:

http://www.biomedcentral.com/1471-2350/13/59/prepub
